# A new Bayesian piecewise linear regression model for dynamic network reconstruction

**DOI:** 10.1186/s12859-021-03998-9

**Published:** 2021-04-26

**Authors:** Mahdi Shafiee Kamalabad, Marco Grzegorczyk

**Affiliations:** 1 Department of Methodology and Statistics, Tilburg School of Social and Behavioral Sciences, Tilburg University, Prof. Cobbenhagenlaan 225, 5037 DB Tilburg, The Netherlands; 2 Jheronimus Academy of Data Science, Sint Janssingel 92, 5211 DA ’s-Hertogenbosch, The Netherlands; 3 Bernoulli Institute, Groningen University, Nijenborgh 9, 9747 AG Groningen, The Netherlands

**Keywords:** Bayesian piece-wise linear regression, Gene regulatory networks, Network reconstruction, Segment-wise parameter coupling

## Abstract

**Background:**

Linear regression models are important tools for learning regulatory networks from gene expression time series. A conventional assumption for non-homogeneous regulatory processes on a short time scale is that the network structure stays constant across time, while the network parameters are time-dependent. The objective is then to learn the network structure along with changepoints that divide the time series into time segments. An uncoupled model learns the parameters separately for each segment, while a coupled model enforces the parameters of any segment to stay similar to those of the previous segment. In this paper, we propose a new consensus model that infers for each individual time segment whether it is coupled to (or uncoupled from) the previous segment.

**Results:**

The results show that the new consensus model is superior to the uncoupled and the coupled model, as well as superior to a recently proposed generalized coupled model.

**Conclusions:**

The newly proposed model has the uncoupled and the coupled model as limiting cases, and it is able to infer the best trade-off between them from the data.

**Supplementary Information:**

The online version supplementary material available at 10.1186/s12859-021-03998-9.

## Background

Non-homogeneous dynamic Bayesian networks have become a popular tool for learning the structures of cellular regulatory networks from gene expression and protein concentration data. The traditional (homogeneous) dynamic Bayesian network models assume the network parameters to stay constant across time. This can lead to biased results and wrong conclusions, as cellular regulatory processes can change in time. It was therefore proposed to combine dynamic Bayesian network models with Bayesian changepoint processes, see, e.g., [1–3]. Then a multiple changepoint process is used to divide the temporal data into disjoint segments, and the data within each segment are modelled by linear regression models. For most cellular processes on a short time scale it is not realistic to assume that the network structure changes over time. The network structure is therefore usually assumed to stay unchanged and only the network parameters are assumed to time-varying. As a motivation for this assumption consider a gene regulatory network, in which an edge from gene $Z_i$ to gene $Z_j$, $Z_i$ $\rightarrow$ $Z_j$, typically would indicate that gene $Z_i$ codes for a transcription factor that can bind to the promoter of gene $Z_j$, so that $Z_j$’s transcription is initiated. The ability to bind to the promoter (= the edge connection) is unlikely to change within a short time period, whereas the extent of binding (= the network interaction parameter) can undergo quick temporal changes. Regarding our two real-life applications to *S. cerevisiae* (yeast) and *A. thaliana* (plant) gene expression data, the assumption of a fixed network structure therefore seems more faithful.

The uncoupled model, akin to the models proposed by Lèbre et al. [1] and Dondelinger et al. [3], learns the segment-specific network parameters for each segment separately. To allow for information-sharing with respect to the network parameters, models with globally [4] and sequentially [5] coupled network parameters were proposed. As sequential information-sharing seems more suitable for temporal time segments, we focus here on the sequential coupling. The underlying idea is that the network parameters of each segment should be enforced to stay similar to those of the previous segment. Grzegorczyk and Husmeier [5] proposed a coupled model, in which the posterior expectations of the network parameters of segment *h* are used as prior expectations for the next segment $h+1$. The strength of the coupling, i.e. the variance of the network parameter priors, is regulated by a coupling parameter. Although it was shown that this is very useful for applications where the network parameters stay similar over time, the fully coupled model has the drawback that it enforces coupling and does not feature any possibility for uncoupling. In this paper we therefore propose a partially segment-wise coupled model, which can be seen as a consensus model between the uncoupled and the fully coupled model. Discrete binary indicator variables $\delta _h$ indicate for each segment *h* whether it is coupled to the previous segment ($\delta _h=1$) or uncoupled from it ($\delta _h=0$). Along with the network structure and the data segmentation the values of those indicator variables are inferred from the data. The new partially coupled model reaches the original models in the limit: If it couples all segments ($\delta _h=1$ for all $h>2$), it becomes the fully coupled model. If it uncouples all segments ($\delta _h=0$ for all *h*), it becomes the uncoupled model.

In our earlier work [6] we have proposed a new generalized fully coupled model. While the fully coupled model from [5] couples all neighbouring segments $(h-1,h)$ with the same coupling strength $\lambda \in \mathbb {R}^{+}$, the generalised (fully) coupled model from [6] uses for each pair of neighbouring segments $(h-1,h)$ a segment-specific coupling strength parameter $\lambda _h\in \mathbb {R}^{+}$. This leads to a higher model flexibility, but like the coupled model the generalized coupled model still does not allow for uncoupling. In our comparative evaluation study, we will compare the new partially coupled model with the three competing models: the uncoupled model, the (fully) coupled model, and the generalized (fully) coupled model.

In recent works alternative model refinements have been proposed [7, 8]. These models distinguish coupled from uncoupled network edges rather than distinguishing coupled from uncoupled time segments. The partially non-homogeneous model from Shafiee Kamalabad et al. [7] builds on the idea that only some network parameters (i.e some edges) might be subject to changes, while other network parameters (i.e. edges) might stay constant. The model has been designed for analysing data that have been measured under different experimental conditions, so that it does not allow the segmentation of a time series to be inferred. The non-homogeneous model from Shafiee Kamalabad and Grzegorczyk [8] distinguishes between two groups of edges: (i) edges that are fully coupled among all segments and (ii) edges that are uncoupled among all segments. The new model that we propose here is conceptual related, but complementary in that it replaces the concept of partially coupled edges by the concept of partially coupled time segments.

We note that network reconstruction is a topical research field in the computational biology literature and that many different network reconstruction approaches have been proposed over the years. However, most of the proposed models do not focus on non-homogeneous regulatory processes but rely on a homogeneity of the regulatory processes. For some applications this assumption of homogeneity can be too restrictive; compare, e.g., our data applications. In response to one of the reviewers of our paper, we here briefly discuss a few recently proposed network reconstruction methods. Vignes et al. [9] investigated and compared a wide variety of methods, ranging from Bayesian networks to penalised linear regression based models and proposed a meta-analysis based on Fisher’s Inverse Chi-Square meta-test for combining different approaches. Huang et al. [10] proposed to apply Bayesian model averaging for linear regression methods. The method uses a closed form solution to compute the edge posterior probabilities within a hybrid framework of Bayesian model averaging and linear regression. Xing et al. [11] proposed a Candidate Auto Selection algorithm based on the pairwise mutual information and breakpoint detection. With a greedy search algorithm it is searched for the best network topology. Unlike the above mentioned models, Fan et al. [12] propose to impose a prior on the topology information in their inference process. Incorporating this prior information can partially compensate for the lack of reliable data. They then developed a Bayesian group lasso with spike and slab prior approach based on non-parametric models. Xu et al. [13] propose to employ a series of linear regression problems to model the relationship between the network nodes. They use an efficient variational Bayes method for optimization and inference of the unknown network parameters.

## Methods

### Learning dynamic networks with time-varying parameters

Consider *N* random variables $Z_1,\ldots ,Z_{N}$ that are the nodes of a network. Let $\mathbf{D }$ denote an *N*-by-$(T+1)$ data matrix, whose *N* rows correspond to the variables and whose $T+1$ columns correspond to time points $t=1,\ldots ,T+1$. The element in the *i*th row and *t*th column, $\mathbf{D }_{i,t}$, is the value of $Z_i$ at time point *t*. For temporal data it is typically assumed that the regulatory interactions are subject to a lag of one time point. For example, an edge $Z_i\rightarrow Z_j$ indicates that $\mathbf{D }_{j,t+1}$ ($Z_j$ at $t+1$) depends on $\mathbf{D }_{i,t}$ ($Z_i$ at *t*). The variable $Z_i$ is then called a parent (node) of $Z_j$.

Because of the lag, there is no need for any acyclicity constraint, and for each node $Z_j$ ($j=1,\ldots ,N$) the parent nodes can be learned separately. This has computational advantages, since the ‘network learning task’ can be separated into *N* independent ‘parent learning tasks’. Henceforth, when a computer cluster is available, the *N* parent sets can be learned in parallel, so that the inference algorithms scale-up well.

A popular method is to apply linear regression, where $Y:=Z_j$ is the response and $\{Z_1,\ldots ,Z_{j-1},Z_{j+1},\ldots ,Z_{N}\}=:\{X_1,\ldots ,X_{n}\}$ are potential covariates (with $n:=N-1$). Because of the lag, $T+1$ time points yield *T* observations for the linear regression model. Each observation $\mathcal {D}_t$ $(t\in \{1,\ldots ,T\})$ consists of a response value $Y=\mathbf{D }_{j,t+1}$ and the shifted covariate values: $X_1=\mathbf{D }_{1,t},\ldots ,X_{j-1}=\mathbf{D }_{j-1,t},X_{j}=\mathbf{D }_{j+1,t},\ldots ,X_{n}=\mathbf{D }_{N,t}$, where $n=N-1$.

Having inferred a covariate set $\varvec{\pi }_j$ for each $Z_j$, a network is built by merging the covariate sets: $\mathcal {G}:=\{\varvec{\pi }_1,\ldots ,\varvec{\pi }_N\}$. There is the edge $Z_i\rightarrow Z_j$ in $\mathcal {G}$ if and only if $Z_i\in \varvec{\pi }_j$.

As the same linear regression approaches are used for each $Z_j$, we describe the models using a general terminology: Let *Y* be the response and let $X_1,\ldots ,X_n$ be the covariates of the linear regression model.

To allow for time-dependent regression coefficients, a piece-wise linear regression model can be used. Changepoints $\varvec{\tau }:=\{\tau _1,\ldots ,\tau _{H-1}\}$ with $1\le \tau _h< T$ divide the observations $\mathcal {D}_1,\ldots ,\mathcal {D}_T$ into disjoint segments $h=1,\ldots ,H$ containing $T_1,\ldots ,T_H$ consecutive data points, so that: $\sum T_h = T$. Observation $\mathcal {D}_t$ ($1\le t\le T$) belongs to segment *h* if $\tau _{h-1}< t\le \tau _h$, where $\tau _0 := 1$ and $\tau _H := T$ are two pseudo changepoints.

We assume all covariate sets $\varvec{\pi }\subset \{X_1,\ldots ,X_n\}$ with up to $\mathcal {F}=3$ covariates to be equally likely a priori, $p(\varvec{\pi })=c$, while parent sets with more than $\mathcal {F}$ covariates get a zero prior probability (‘fan-in restriction’). Further we assume that the distance between changepoints is geometrically distributed with hyperparameter $p\in (0,1)$, so that

$$\begin{aligned} p(\varvec{\tau }) = (1-p)^{\tau _H-\tau _{H-1}-1}\cdot \prod _{h=1}^{H-1} p\cdot (1-p)^{\tau _h-\tau _{h-1}-1} = (1-p)^{(T-1)-(H-1)} \cdot p^{H-1} \end{aligned}$$

With $\mathbf{y }=\mathbf{y }_{\varvec{\tau }} : =\{\mathbf{y }_1,\ldots ,\mathbf{y }_H\}$ being the set of segment-specific response vectors, implied by the changepoint set $\varvec{\tau }$, the posterior distribution takes the form:

1 $$\begin{aligned} p\left( \varvec{\pi },\varvec{\tau },\varvec{\theta }|\mathbf{y }\right) \propto p(\varvec{\pi }) \cdot p(\varvec{\tau }) \cdot p(\varvec{\theta }|\varvec{\pi },\varvec{\tau }) \cdot p\left( \mathbf{y }|\varvec{\pi },\varvec{\tau },\varvec{\theta }\right) \end{aligned}$$

where $\varvec{\theta }= \varvec{\theta }(\varvec{\pi },\varvec{\tau })$ denotes the set of all model parameters, including segment-specific parameters as well as parameters that are shared among segments.

In the following subsections we assume $\varvec{\pi }\subset \{X_1,\ldots ,X_n\}$ and the segmentation $\mathbf{y }=\{\mathbf{y }_1,\ldots ,\mathbf{y }_H\}$, induced by $\varvec{\tau }$, to be fixed, and we do not make $\varvec{\pi }$ and $\varvec{\tau }$ explicit anymore. Without loss of generality, we assume that $\varvec{\pi }$ contains the first *k* covariates: $\varvec{\pi }:=\{X_1,\ldots ,X_k\}$. For fixed $\varvec{\pi }$ and $\varvec{\tau }$, Eq. (1) reduces to:

$$\begin{aligned} p(\varvec{\theta }|\mathbf{y }) \propto p(\varvec{\theta }) \cdot p(\mathbf{y }|\varvec{\theta }) \end{aligned}$$

### A generic Bayesian piece-wise linear regression model

**Fig. 1:**
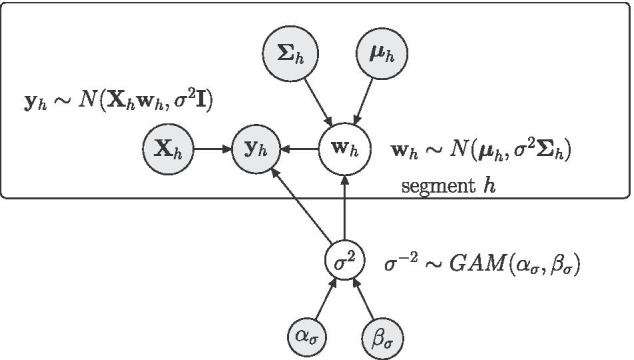
Graphical representation of the generic model. Parameters that have to be inferred are represented by white circles. The data and the fixed hyperparameters are represented by grey circles. Circles within the plate are specific for segment *h*

Consider a Bayesian linear regression model, where *Y* is the response and $X_1,\ldots ,X_k$ are the covariates. We assume that *T* observations $\mathcal {D}_1,\ldots ,\mathcal {D}_T$ have been made at equidistant time points and that the data can be subdivided into disjoint segments $h\in \{1,\ldots ,H\}$, where segment *h* contains $T_h$ data points and has a segment-specific regression coefficient vector $\varvec{{w}}_{h}$. Let $\mathbf{y }_h$ be the response vector and $\mathbf{X }_{h}$ be the design matrix for segment *h*, where each $\mathbf{X }_{h}$ includes a first column of 1’s for the intercept. For each segment $h=1,\ldots ,H$ we assume a Gaussian likelihood:

2 $$\begin{aligned} \mathbf{y }_{h}|\left( \varvec{{w}}_{h},\sigma ^2\right) \sim \mathcal {N}\left( \mathbf{X }_{h} \varvec{{w}}_{h} , \sigma ^2 \mathbf{I }\right) \end{aligned}$$

where $\mathbf{I }$ is the identity matrix, and $\sigma ^{2}$ is a noise variance parameter that is shared among all segments. We impose an inverse Gamma prior on $\sigma ^2$, $\sigma ^{-2}\sim GAM(\alpha _{\sigma },\beta _{\sigma })$, and we assume that the vectors $\varvec{{w}}_{h}$ have Gaussian priors:

3 $$\begin{aligned} \varvec{{w}}_{h}|\left( \varvec{\mu }_h,\varvec{\Sigma }_h,\sigma ^2\right) \sim \mathcal {N}\left( \varvec{\mu }_h, \sigma ^2 \varvec{\Sigma }_h \right) \end{aligned}$$

where $\varvec{\mu }_h$ is a (k+1)-dimensional vector, and $\varvec{\Sigma }_h$ is a positive definite $(k+1)$-by-$(k+1)$ matrix. Re-using the parameter $\sigma ^2$ in Eq. (3), yields a fully-conjugate prior in both $\varvec{{w}}_h$ and $\sigma ^2$ (see, e.g., Sections 3.3 and 3.4 in Gelman [14]). Figure 1 shows a graphical model representation of this generic model. For notational convenience we define:

$$\begin{aligned} \varvec{\theta }:= \left\{ \varvec{\mu }_1,\ldots ,\varvec{\mu }_H;\varvec{\Sigma }_1,\ldots ,\varvec{\Sigma }_H\right\} \end{aligned}$$

**Fig. 2:**
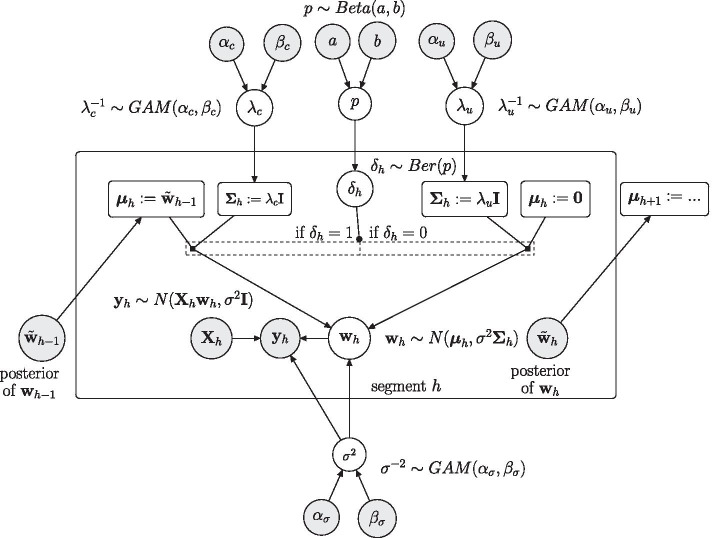
Graphical representation of the new partially coupled model (M3). Parameters that have to be inferred are represented by white circles. The data and the fixed hyperparameters are represented by grey circles. The two rectangles indicate definitions, which depend on the parent nodes. Circles and definitions within the plate are segment-specific. For each segment the model infers if the prior for $\varvec{{w}}_h$ is coupled to ($\delta _h=1$) or uncoupled from ($\delta _h=0$) the preceding segment $h-1$

**Fig. 3:**
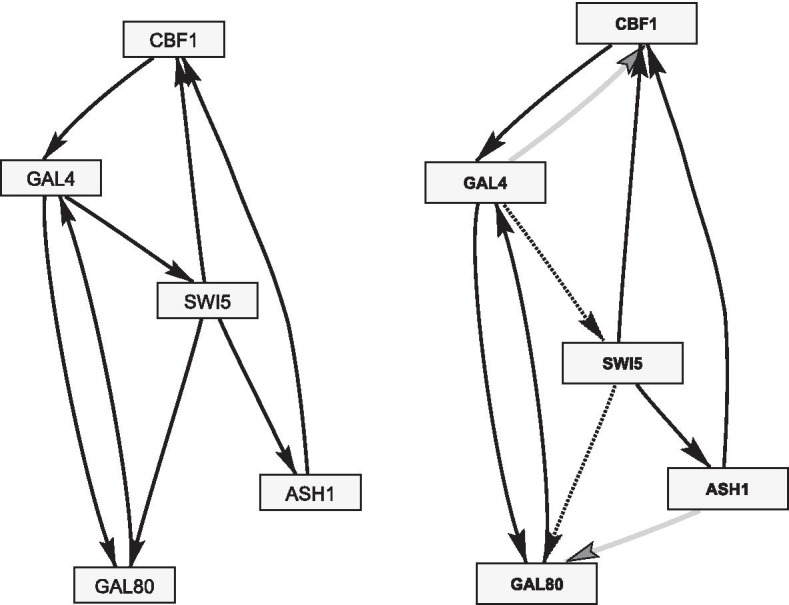
Yeast networks. Left: the true yeast network with $N=5$ nodes and $M=8$ edges. Right: yeast network prediction obtained with model M3. The grey (dotted) edges correspond to false positives (negatives)

**Fig. 4:**
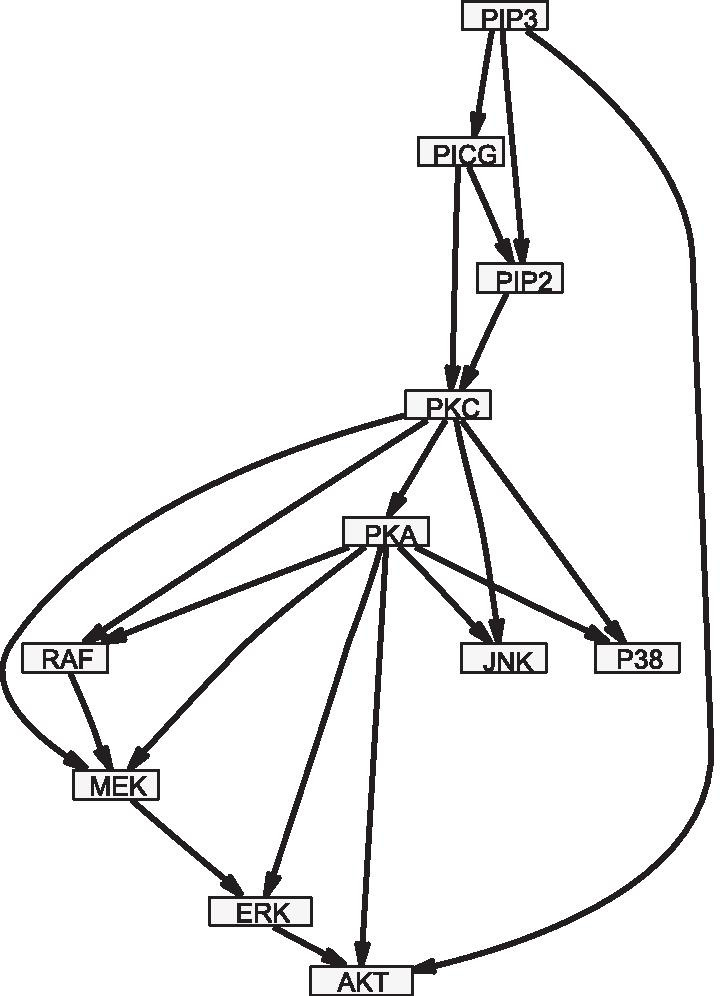
RAF network. RAF pathway with $N=11$ nodes and $M=20$ edges

**Fig. 5:**
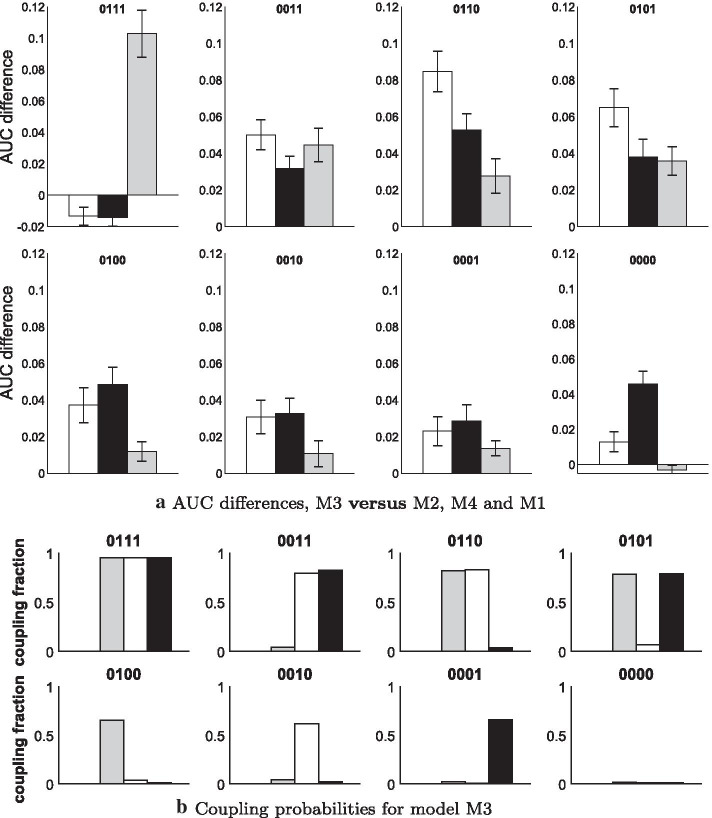
Results for synthetic RAF pathway data. We distinguish 8 coupling scenarios $(\delta _1=0,\delta _2,\delta _3,\delta _4)$. **a** Each histogram has three bars for the average AUC differences between the partially coupled model (M3) and the other models: ‘M3 versus M2 [= Coupled]’ (white), ‘M3 versus M4 [= Generalised]’ (black), and ‘M3 versus M1 [= Uncoupled]’ (grey). The error bars indicate t-test confidence intervals. **b** Diagnostic for the partially coupled model (M3): The bars give the posterior probabilities $p(\delta _h=1|\mathcal {D})$ that segment *h* is coupled to $h-1$ ($h=2,3,4$)

The full conditional distribution of $\varvec{{w}}_h$ is (cp. Section 3.3 in [15]):

4 $$\begin{aligned} \varvec{{w}}_{h}|\left( \mathbf{y }_h,\sigma ^2,\varvec{\theta }\right) \sim \mathcal {N}\left( \left[ \varvec{\Sigma }_h^{-1} + \mathbf{X }_h^{\mathsf{T}} \mathbf{X }_h\right] ^{-1} \left( \varvec{\Sigma }_h^{-1} \varvec{\mu }_h + \mathbf{X }_h^{\mathsf{T}} \mathbf{y }_h\right) ,\sigma ^2 \left( \varvec{\Sigma }_h^{-1} + \mathbf{X }_h^{\mathsf{T}} \mathbf{X }_h\right) ^{-1} \right) \end{aligned}$$

and the segment-specific marginal likelihoods with $\varvec{{w}}_h$ integrated out are:

5 $$\begin{aligned} \mathbf{y }_h|\left( \sigma ^2,\varvec{\theta }\right) \sim \mathcal {N}\left( \mathbf{X }_h \varvec{\mu }_h, \sigma ^2 \mathbf{C }_h(\varvec{\theta }) \right) \end{aligned}$$

where $\mathbf{C }_h(\varvec{\theta }):= \mathbf{I } + \mathbf{X }_h \varvec{\Sigma }_h \mathbf{X }_h^{\mathsf{T}}$ (cp. Section 3.3 in [15]). From Eq. (5) we get:

$$\begin{aligned} p\left( \sigma ^{2}|\mathbf{y },\varvec{\theta }\right) \propto p(\sigma ^2) \cdot \prod _{h=1}^H p\left( \mathbf{y }_h|\sigma ^{2},\varvec{\theta }\right) = \left( \sigma ^{-2}\right) ^{a_{\sigma }+\frac{1}{2} \cdot T-1} e^{-\sigma ^{-2} \left( b_{\sigma } + \frac{1}{2} \cdot \Delta ^2(\varvec{\theta })\right) } \end{aligned}$$

where $\mathbf{y} :=\{\mathbf{y }_1,\ldots ,\mathbf{y }_H\}$ and $\Delta ^2(\varvec{\theta }) := \sum _{h=1}^{H}(\mathbf{y }_{h}- \mathbf{X }_h \varvec{\mu }_h)^{\mathsf{T}} \mathbf{C }_h(\varvec{\theta })^{-1}(\mathbf{y }_{h}- \mathbf{X }_h \varvec{\mu }_h)$. The shape of $p(\sigma ^{2}|\mathbf{y },\varvec{\theta })$ implies:

6 $$\begin{aligned} \sigma ^{-2}|(\mathbf{y },\varvec{\theta }) \sim GAM\left( \alpha _{\sigma } + \frac{1}{2} \cdot T, \beta _{\sigma }+ \frac{1}{2}\cdot \Delta ^2(\varvec{\theta }) \right) \end{aligned}$$

For the marginal likelihood, with $\varvec{{w}}_h$ ($h=1,\ldots ,H$) and $\sigma ^2$ integrated out, we apply the rule from Section 2.3.7 of Bishop [15]:

7 $$\begin{aligned} p(\mathbf{y }|\varvec{\theta }) = \frac{\Gamma (\frac{T}{2}+a_{\sigma })}{\Gamma (a_{\sigma })} \cdot \frac{\pi ^{-T/2}\cdot (2b_{\sigma })^{a_{\sigma }}}{ \left( \prod \limits _{h=1}^{H}\det ( \mathbf{C} _h(\varvec{\theta }) )\right) ^{1/2}} \cdot \left( 2b_{\sigma } + \Delta ^2(\varvec{\theta })\right) ^{-\left(\frac{T}{2} + a_{\sigma }\right)} \end{aligned}$$

When all parameters in $\varvec{\theta }$ are fixed, the marginal likelihood of the piece-wise linear regression model can be computed in closed form. In typical models the (hyper-)hyperparameters in $\varvec{\theta }$ depend on hyperparameters with their own hyperprior distributions. From now on we will only include the free hyperparameters in $\varvec{\theta }$. In the following subsections we describe four possible model instantiations, namely: the uncoupled model (M1), the coupled model (M2), the newly proposed partially coupled model (M3), and the generalized coupled model (M4). In the forthcoming subsections we will introduce further mathematical symbols. For convenience, Table 1 lists the mathematical symbols that we will use in this paper.

**Table 1:** List of mathematical symbols

Symbol	Description	Prior distribution
*N*	Total number of nodes (genes)	–
*n*	Number of potential parent nodes, here $n=N-1$	–
*h*	Data segment *h*	–
*H*	Total number of data segments	–
*k*	Number of covariates in covariate set	–
*t*	Data point *t*	–
$\sigma ^2$	Noise variance parameter	$\sigma ^{-2}\sim GAM(\alpha _{\sigma },\beta _{\sigma })$
$\lambda _c$	Coupling strength parameter, $h>1$	$\lambda _c^{-1}\sim GAM(\alpha _{c},\beta _{c})$
$\lambda _u$	SNR parameter, $h=1$	$\lambda _u^{-1}\sim GAM(\alpha _{u},\beta _{u})$
$\lambda _h$	*h*th coupling strength parameter (M4 model)	$\lambda _h^{-1}\sim GAM(\alpha _{c},\beta _{c})$
$\delta _h$	*h*th coupling indicator variable (M3 model)	$\delta _h\sim BER(\text{ p})$, $\text{ p }\sim BETA(a,b)$
*T*	Total number of data points	–
$T_h$	Number of data points in segment *h*	–
$D_i$	*i*th data point	–
$Z_i$	*i*th network node	–
${\varvec{\pi }_{i}}$	Parent (covariate) set of *i*th node, $Z_i$	$p(|\varvec{\pi }|<=3)=c$, $p(|\varvec{\pi }|>3)=0$
$\varvec{\tau }$	Changepoint set	$p(\varvec{\tau }) = (1-p)^{(T-1)-(H-1)} \cdot p^{H-1}$
$\tau _h$	Changepoint *h*	–
$X_i$	*i*th covariate	–
$\mathbf{X }_{h}$	Design matrix of segment *h*	–
$\mathbf{y }_{h}$	Response vector of segment *h*	$\mathbf{y }_{h}|(\varvec{{w}}_{h},\sigma ^2) \sim \mathcal {N}(\mathbf{X }_{h} \varvec{{w}}_{h} , \sigma ^2 \mathbf{I })$
$\varvec{{w}}_{h}$	Regression coefficient vector of segment *h*	$\varvec{{w}}_{h}|(\varvec{\mu }_h,\varvec{\Sigma }_h,\sigma ^2) \sim \mathcal {N}(\varvec{\mu }_h, \sigma ^2 \varvec{\Sigma }_h )$
$\tilde{\varvec{{w}}_{h-1}}$	Posterior expectation of $\varvec{{w}}_{h-1}$	–

### Model M1: the uncoupled model

A standard approach, akin to the models of Lèbre et al. [1] and Dondelinger et al. [3], is to set $\varvec{\mu }_h=\mathbf{0 }$ and to assume that the matrices $\varvec{\Sigma }_h$ are diagonal matrices $\varvec{\Sigma }_h=\lambda _u \mathbf{I }$, where the parameter $\lambda _u\in \mathbf {R}^+$ is shared among segments and assumed to be inverse Gamma distributed, $\lambda _u^{-1} \sim GAM(\alpha _u,\beta _u)$. In the supplementary material we provide a graphical model representation of the uncoupled model (M1). Using the notation of the generic model, we have:

8 $$\begin{aligned} \varvec{\theta }=\{\lambda _u\}, \;\;\;\; \mathbf{C} _{h}(\lambda _u)=\mathbf{I } + \lambda _u \mathbf{X }_h \mathbf{X }_h^{\mathsf{T}}, \;\;\;\; \Delta ^2(\lambda _u) := \sum _{h=1}^{H}\mathbf{y }_{h}^{\mathsf{T}} \mathbf{C }_h(\lambda _u)^{-1}\mathbf{y }_{h} \end{aligned}$$

For the posterior distribution of the uncoupled model we have:

9 $$\begin{aligned} p\left( \varvec{{w}},\sigma ^2,\lambda _u|\mathbf{y }\right) \propto p\left( \sigma ^2\right) \cdot p\left( \lambda _u\right) \cdot \prod _{h=1}^H p\left( \varvec{{w}}_h|\sigma ^2, \lambda _u\right) \cdot \prod _{h=1}^H p\left( \mathbf{y }_h|\sigma ^2, \varvec{{w}}_h\right) \end{aligned}$$

where $\varvec{{w}}:=\{\varvec{{w}}_1,\ldots , \varvec{{w}}_H \}$. From Eq. (9) it follows for the full conditional distribution of $\lambda _u$:

$$\begin{aligned} p\left( \lambda _u|\mathbf{y} ,\varvec{{w}},\sigma ^2\right)&\propto {} p\left( \lambda _u\right) \cdot \prod \limits _{h=1}^H p\left( \varvec{{w}}_h|\sigma ^2,\lambda _u\right) \\&\propto {} \left( \lambda _u^{-1}\right) ^{ a_u + \frac{H\cdot (k+1)}{2}} \cdot \exp \left\{ -\lambda _u^{-1}\left( b_u + \frac{1}{2} \sigma ^{-2} \sum _{h=1}^H \varvec{{w}}_h^{\mathsf{T}}\varvec{{w}}_h\right) \right\} \end{aligned}$$

and the shape of the latter density implies:

10 $$\begin{aligned} \lambda _u^{-1}|\left( \mathbf{y} ,\varvec{{w}},\sigma ^2\right) \sim GAM\left( \alpha _u + \frac{H\cdot (k+1)}{2}, \beta _u + \frac{1}{2} \sigma ^{-2} \sum _{h=1}^H \varvec{{w}}_h^{\mathsf{T}}\varvec{{w}}_h \right) \end{aligned}$$

Since the full conditional distribution of $\lambda _u$ depends on $\sigma ^2$ and $\varvec{{w}}$, those parameters have to be sampled first. From Eq. (6) a value of $\sigma ^2$ can be sampled via a collapsed Gibbs-sampling step, with the $\varvec{{w}}_h$’s being integrated out. Subsequently, given $\sigma ^2$, Eq. (4) can be used to sample the vectors $\varvec{{w}}_h$’s. Finally, for each $\lambda _u$ sampled from Eq. (10) the marginal likelihood, $p(\mathbf{y }|\lambda _u)$, can be computed by plugging in the expressions from Eq. (8) into Eq. (7).

### Model M2: the (fully) coupled model

The (fully) coupled model, proposed by Grzegorczyk and Husmeier [5], uses the posterior expectation of $\varvec{{w}}_{h-1}$ as prior expectation for $\varvec{{w}}_{h}$. Only the first segment $h=1$ has an uninformative prior:

11 $$\begin{aligned} \varvec{{w}}_{h} \sim {\left\{ \begin{array}{ll} \mathcal {N}\left( \mathbf{0 }, \sigma ^2 \lambda _u \mathbf{I } \right) &{}\quad \text{ if } h=1 \\ \mathcal {N}\left( \tilde{\varvec{{w}}}_{h-1}, \sigma ^2 \lambda _c \mathbf{I } \right) &{}\quad \text{ if } h> 1 \end{array}\right. } \end{aligned}$$

where $\tilde{\varvec{{w}}}_{h-1}$ is the posterior expectation of $\varvec{{w}}_{h-1}$ (cp. Eq. (4)):

$$\begin{aligned} \tilde{\varvec{{w}}}_{h-1}:= {\left\{ \begin{array}{ll} [\varvec{\Sigma }_{1}^{-1} + \mathbf{X }_{1}^{\mathsf{T}} \mathbf{X }_{1}]^{-1} \left( \mathbf{X }_{1}^{\mathsf{T}} \mathbf{y }_{1}\right) &{}\quad \text{ if } h=2 \\ \left[ \varvec{\Sigma }_{h-1}^{-1} + \mathbf{X }_{h-1}^{\mathsf{T}} \mathbf{X }_{h-1}\right] ^{-1} \left( \lambda _c^{-1} \tilde{\varvec{{w}}}_{h-2} + \mathbf{X }_{h-1}^{\mathsf{T}} \mathbf{y }_{h-1}\right) &{}\quad \text{ if } h>2 \end{array}\right. } \end{aligned}$$

The parameter $\lambda _{c}$ has been called the ’*coupling parameter*’ and it has been assumed that it has an inverse Gamma prior distribution, $\lambda _c^{-1}\sim GAM(\alpha _{c},\beta _{c})$. Using the notation from the generic model (see Fig. 1), we note that Eq. (11) corresponds to:

$$\begin{aligned} \varvec{\mu }_{h} = {\left\{ \begin{array}{ll} \mathbf{0 } &{}\quad \text{ if } h=1 \\ \tilde{\varvec{{w}}}_{h-1} &{}\quad \text{ if } h> 1 \end{array}\right. },\;\;\;\; \varvec{\Sigma }_{h} = {\left\{ \begin{array}{ll} \lambda _u \mathbf{I } &{}\quad \text{ if } h=1 \\ \lambda _c \mathbf{I } &{}\quad \text{ if } h> 1 \end{array}\right. },\\ \mathbf{C} _{h}(\varvec{\theta }) = {\left\{ \begin{array}{ll} \mathbf{I } + \lambda _u \mathbf{X }_h \mathbf{X }_h^{\mathsf{T}} &{}\quad \text{ if } h=1 \\ \mathbf{I } + \lambda _c \mathbf{X }_h \mathbf{X }_h^{\mathsf{T}} &{}\quad \text{ if } h> 1 \end{array}\right. }, \;\;\;\; \varvec{\theta }= \{\lambda _u,\lambda _c\}, \quad \Delta ^2(\varvec{\theta }) = \sum _{h=1}^{H}\left( \mathbf{y }_{h}- \mathbf{X }_h \tilde{\varvec{{w}}}_{h-1}\right) ^{\mathsf{T}} \mathbf{C }_h(\varvec{\theta })^{-1}\left( \mathbf{y }_{h}- \mathbf{X }_h \tilde{\varvec{{w}}}_{h-1}\right) \end{aligned}$$

with $\tilde{\varvec{{w}}}_{0}:=\mathbf{0 }$, $\lambda _u^{-1}\sim GAM(\alpha _u,\beta _u)$ and $\lambda _c^{-1}\sim GAM(\alpha _c,\beta _c)$. As $\tilde{\varvec{{w}}}_{h-1}$ is treated like a fixed hyperparameter when used as input for segment *h*, we exclude the parameters $\tilde{\varvec{{w}}}_{1},\ldots ,\tilde{\varvec{{w}}}_{H-1}$ from $\varvec{\theta }$.

In the supplementary material we provide a graphical model representation of the coupled M2 model. For the posterior we have:

12 $$\begin{aligned} p\left( \varvec{{w}},\sigma ^2,\lambda _u,\lambda _c|\mathbf{y }\right)\propto p\left( \sigma ^2\right) \cdot p\left( \lambda _u\right) \cdot p\left( \lambda _c\right) \cdot p\left( \varvec{{w}}_1|\sigma ^2, \lambda _u\right) \cdot \nonumber \\&\prod _{h=2}^H p\left( \varvec{{w}}_h|\sigma ^2, \lambda _c\right) \cdot \prod _{h=1}^H p\left( \mathbf{y }_h|\sigma ^2, \varvec{{w}}_h\right) \end{aligned}$$

In analogy to the derivations in the previous subsection one can derive (cp. [5]):

13 $$\begin{aligned} \lambda _u^{-1}|\left( \mathbf{y },\varvec{{w}},\sigma ^2,\lambda _c\right)\sim GAM\left( \alpha _u + \frac{1\cdot (k+1)}{2}, \beta _u + \frac{1}{2} \sigma ^{-2} D_u^2\right) \end{aligned}$$

14 $$\begin{aligned} \lambda _c^{-1}|(\mathbf{y} ,\varvec{{w}},\sigma ^2,\lambda _u)\sim GAM\left( \alpha _c + \frac{(H-1)\cdot (k+1)}{2}, \beta _c + \frac{1}{2} \sigma ^{-2} D_c^2 \right) \end{aligned}$$

where $$D_u^2 := \varvec{{w}}_{1}^{\mathsf{T}} \varvec{{w}}_{1}$$ and $$D_c^2 := \sum _{h=2}^{H}(\varvec{{w}}_{h}- \tilde{\varvec{{w}}}_{h-1})^{\mathsf{T}} (\varvec{{w}}_{h}- \tilde{\varvec{{w}}}_{h-1})$$.

For each $\varvec{\theta }=\{\lambda _u,\lambda _c\}$ the marginal likelihood, $p(\mathbf{y }|\lambda _u,\lambda _c)$, can be computed by plugging the expressions $\mathbf{C} _h(\varvec{\theta })$ and $\Delta ^2(\varvec{\theta })$ into Eq. (7).

### Model M3: the new partially segment-wise coupled model

We propose a new ‘consensus’ model between the M1 and the M2 model. The new model (M3) allows each segment $h>1$ either to coupled top or to uncouple from the preceding segment $h-1$. We use an uninformative prior for the first segment $h=1$, and for all segments $h>1$ we introduce a binary variable $\delta _h$ which indicates whether segment *h* is coupled to ($\delta _h=1$) or uncoupled from ($\delta _h=0$) the preceding segment $h-1$:

15 $$\begin{aligned} \varvec{{w}}_{h} \sim {\left\{ \begin{array}{ll} \mathcal {N}\left( \mathbf{0 }, \sigma ^2 \lambda _u \mathbf{I } \right) &{}\quad \text{ if } h=1 \\ \mathcal {N}\left( \delta _h \cdot \tilde{\varvec{{w}}}_{h-1}, \sigma ^2 \lambda _c^{\delta _{h}} \lambda _u^{1-\delta _{h}}\mathbf{I } \right) &{}\quad \text{ if } h> 1 \end{array}\right. } \end{aligned}$$

where $\tilde{\varvec{{w}}}_{h-1}$ is the posterior expectation of $\varvec{{w}}_{h-1}$. The new priors from Eq. (15) yield for $h\ge 2$ the following posterior expectations (cp. Eq. (4)):

$$\begin{aligned} \tilde{\varvec{{w}}}_{h-1} = \left( \lambda _c^{-\delta _{h-1}} \lambda _u^{-\left( 1-\delta _{h-1}\right) } \mathbf{I } +\mathbf{X } _{h-1}^{\mathsf{T}} \mathbf{X } _{h-1} \right) ^{-1} \left( \delta _{h-1} \lambda _c^{-1} \tilde{\varvec{{w}}}_{h-2} + \mathbf{X }_{h-1}^{\mathsf{T}} \mathbf{y }_{h-1}\right) \end{aligned}$$

with $\tilde{\varvec{{w}}}_0:=\mathbf{0 }$, $\delta _1:=0$, we have in the generic model notation:

$$\begin{aligned} \varvec{\mu }_{h} = \delta _h \tilde{\varvec{{w}}}_{h-1}, \;\;\;\varvec{\Sigma }_{h} = \lambda _c^{\delta _{h}} \lambda _u^{1-\delta _{h}}\mathbf{I },\;\;\; \varvec{\theta }= \left\{ \lambda _u,\lambda _c,\left\{ \delta _h\right\} _{h \ge 2}\right\},\;\; \mathbf{C} _{h}(\varvec{\theta }) = \mathbf{I } + \lambda _c^{\delta _{h}} \lambda _u^{1-\delta _{h}} \mathbf{X }_h \mathbf{X }_h^{\mathsf{T}} \end{aligned}$$

We assume the binary variables $\delta _2,\ldots ,\delta _H$ to have a Bernoulli prior distributions, $\delta _h\sim BER(\text{ p})$, with a joint hyperparameter $\text{ p }\in [0,1]$ having a Beta hyperprior distribution, $\text{ p }\sim BETA(a,b)$. We note that

- $\delta _h=0$ ($h\ge 2$) gives model M1 with $P(\varvec{{w}}_{h}) = \mathcal {N}(\mathbf{ 0 },\lambda _u \sigma ^2 \mathbf{I })$ for all *h*
- $\delta _h=1$ ($h\ge 2$) gives model M2 with $P(\varvec{{w}}_{h}) = \mathcal {N}(\tilde{\varvec{{w}}}_{h-1}, \lambda _c \sigma ^2 \mathbf{I })$ for $h\ge 2$.
- The new partially segment-wise coupled model infers the variables $\delta _h$ ($h\ge 2$) from the data. It searches for the best trade-off between the models M1 and M2.

A graphical model presentation of the partially coupled model is shown in Fig. 2. For $\delta _h\sim BER(\text{ p})$ with $\text{ p }\sim BETA(a,b)$ the joint marginal density of $\{\delta _h\}_{h\ge 2}$ is:

16 $$\begin{aligned} p\left( \left\{ \delta _h\right\} _{h\ge 2}\right) = \int p(\text{ p}) \prod \limits _{h=2}^H p(\delta _h|\text{p}) \;dp = \frac{\Gamma (a+b)}{\Gamma (a)\Gamma (b)} \cdot \frac{\Gamma \left( a+\sum \limits _{h=2}^H \delta _h\right) \Gamma \left( b + \sum \limits _{h=2}^H \left( 1-\delta _h\right) \right) }{\Gamma \left( a+b+(H-1)\right) } \end{aligned}$$

For the posterior distribution of the partially segment-wise coupled model we get:

$$\begin{aligned} p\left( \varvec{{w}},\sigma ^2,\lambda _u,\lambda _c,\{\delta _h\}_{h\ge 2}|\mathbf{y }\right)\propto\; & {} p\left( \sigma ^2\right) \cdot p\left( \lambda _u\right) \cdot p\left( \lambda _c\right) \cdot p\left( \{\delta _h\}_{h\ge 2}\right) \cdot p\left( \varvec{{w}}_1|\sigma ^2,\lambda _u\right) \\&\cdot \prod _{h=2}^H p\left( \varvec{{w}}_h|\sigma ^2,\lambda _u,\lambda _c,\delta _h\right) \cdot \prod _{h=1}^H p\left( \mathbf{y }_h|\sigma ^2,\varvec{{w}}_h\right) \end{aligned}$$

For the full conditional distributions of $\lambda _u$ and $\lambda _c$ we have:

$$\begin{aligned} p\left( \lambda _u|\mathbf{y} ,\varvec{{w}},\sigma ^2,\lambda _c, \{\delta _h\}_{h\ge 2}\right)\propto\; & p\left( \lambda _u\right) \cdot \prod \limits _{h:\delta _h=0} p\left( \varvec{{w}}_h|\sigma ^2,\lambda _u\right) \\ p\left( \lambda _c|\mathbf{y} ,\varvec{{w}},\sigma ^2,\lambda _u, \{\delta _h\}_{h\ge 2}\right)\propto\; & p\left( \lambda _c\right) \cdot \prod \limits _{h:\delta _h=1} p\left( \varvec{{w}}_h|\sigma ^2,\lambda _c\right) \end{aligned}$$

where $\delta _1:=0$ fixed. And it follows from the shapes of the densities:

$$\begin{aligned} \lambda _u^{-1}|(\mathbf{y} ,\varvec{{w}},\sigma ^2,\lambda _c,\{\delta _h\}_{h\ge 2} )\sim\; & GAM\left( \alpha _u + \frac{H_u\cdot (k+1)}{2}, \beta _u + \frac{1}{2} \sigma ^{-2} D_u^2 \right) \\ \lambda _c^{-1}|(\mathbf{y} ,\varvec{{w}},\sigma ^2,\lambda _u, \{\delta _h\}_{h\ge 2})\sim \;& GAM\left( \alpha _c + \frac{H_c\cdot (k+1)}{2}, \beta _c + \frac{1}{2} \sigma ^{-2} D_c^2 \right) \end{aligned}$$

where $H_c= \sum _h \delta _h$ is the number of coupled segments, $H_u= \sum _h (1-\delta _h)$ is the number of uncoupled segments, so that $H_c+H_u=H$, and

$$\begin{aligned} D_u^2 := \sum \limits _{h:\delta _h=0} \varvec{{w}}_{h}^{\mathsf{T}} \varvec{{w}}_{h}, \;\; D_c^2 := \sum \limits _{h:\delta _h=1}\left( \varvec{{w}}_{h}- \tilde{\varvec{{w}}}_{h-1}\right) ^{\mathsf{T}} \left( \varvec{{w}}_{h}- \tilde{\varvec{{w}}}_{h-1}\right) \end{aligned}$$

For each parameter instantiation $\varvec{\theta }=\{\lambda _u,\lambda _c,\{\delta _h\}_{h\ge 2}\}$ the marginal likelihood, $p(\mathbf{y }|\varvec{\theta })$, can be computed with Eq. (7), where $\mathbf{C} _h(\varvec{\theta })$ was defined above, and

$$\begin{aligned} \Delta ^2(\varvec{\theta }) = \sum _{h=1}^{H}\left( \mathbf{y }_{h}-\delta _h \mathbf{X }_h \tilde{\varvec{{w}}}_{h-1}\right) ^{\mathsf{T}} \left[ \mathbf{I } + \lambda _c^{\delta _{h}} \lambda _u^{1-\delta _{h}}\mathbf{X }_h \mathbf{X }_h^{\mathsf{T}} \right] ^{-1} \left( \mathbf{y }_{h}-\delta _h \mathbf{X }_h \tilde{\varvec{{w}}}_{h-1}\right) \end{aligned}$$

We have for each binary variable $\delta _k$ ($k=2,\ldots ,H$):

$$\begin{aligned} p\left( \delta _k=1|\lambda _u,\lambda _c,\{\delta _h\}_{h\ne k},\mathbf{y }\right) \propto p(\mathbf{y }|\lambda _u,\lambda _c,\{\delta _h\}_{h\ne k},\delta _k=1) \cdot p\left( \{\varvec{\delta }_h\}_{h\ne k},\delta _k=1\right) \end{aligned}$$

so that its full conditional distribution is:

$$\begin{aligned} \delta _k|\left( \lambda _u,\lambda _c,\{\delta _h\}_{h\ne k},\mathbf{y }\right) \sim BER\left( \frac{p\left( \mathbf{y }|\lambda _u,\lambda _c,\{\delta _h\}_{h\ne k},\delta _k=1\right) \cdot p\left( \{\varvec{\delta }_h\}_{h\ne k},\delta _k=1\right) }{\sum \limits _{j=0}^1 p\left( \mathbf{y }|\lambda _u,\lambda _c,\{\delta _h\}_{h\ne k},\delta _k=j\right) \cdot p\left( \{\varvec{\delta }_h\}_{h\ne k},\delta _k=j\right) } \right) \end{aligned}$$

Each $\delta _k$ ($k>1$) can therefore be sampled with a collapsed Gibbs sampling step, where $\{\varvec{{w}}_h\}$, $\sigma ^2$ and $\text{ p }$ have been integrated out.

### Model M4: the generalised (fully) coupled model

In [6] we proposed to generalise the (fully) coupled model (i.e. the M2 model) by introducing a segment-specific coupling parameter $\lambda _h$ for each segment $h>2$. This yields:

17 $$\begin{aligned} \varvec{{w}}_{h} \sim {\left\{ \begin{array}{ll} \mathcal {N}\left( \mathbf{0 }, \sigma ^2 \lambda _u \mathbf{I } \right) &{}\quad \text{ if } h=1 \\ \mathcal {N}\left( \tilde{\varvec{{w}}}_{h-1}, \sigma ^2 \lambda _h \mathbf{I } \right) &{}\quad \text{ if } h> 1 \end{array}\right. } \end{aligned}$$

where $\tilde{\varvec{{w}}}_{h-1}$ is the posterior expectation of $\varvec{{w}}_{h-1}$. For the parameters $\lambda _{h}$ we have assumed that they are inverse Gamma distributed, $\lambda _h^{-1}\sim GAM(\alpha _{c},\beta _{c})$, with hyperparameters $\alpha _c$ and $\beta _c$. In the supplementary material we provide a graphical model representation of the M4 model. Recalling the generic notation and setting $\tilde{\varvec{{w}}}_{0}:=\mathbf{0 }$ and $\lambda _1 := \lambda _u$, Eq. (17) gives:

$$\begin{aligned} \varvec{\mu }_{h} = \tilde{\varvec{{w}}}_{h-1}, \quad \varvec{\Sigma }_{h} = \lambda _h \mathbf{I }, \quad \mathbf{C} _{h}(\varvec{\theta }) = \mathbf{I } + \lambda _h \mathbf{X }_h \mathbf{X }_h^{\mathsf{T}}, \quad \varvec{\theta }= \{\lambda _u, \{\lambda _h\}_{h\ge 2}\}, \\ \text{ and } \; \Delta ^2(\varvec{\theta }) = \sum _{h=1}^{H}\left( \mathbf{y }_{h}- \mathbf{X }_h \tilde{\varvec{{w}}}_{h-1}\right) ^{\mathsf{T}} \mathbf{C }_h(\varvec{\theta })^{-1}\left( \mathbf{y }_{h}- \mathbf{X }_h \tilde{\varvec{{w}}}_{h-1}\right) \end{aligned}$$

For the posterior we have:

18 $$\begin{aligned} p\left( \varvec{{w}},\sigma ^2,\lambda _u,\{\lambda _h\}_{h\ge 2}|\mathbf{y }\right)\propto \;& p\left( \sigma ^2\right) \cdot p(\lambda _u) \cdot \left( \prod _{h=2}^H p(\lambda _h) \right) \nonumber \\&\cdot p\left( \varvec{{w}}_1|\sigma ^2, \lambda _u\right) \cdot \prod _{h=2}^H p\left( \varvec{{w}}_h|\sigma ^2, \lambda _h\right) \cdot \prod _{h=1}^H p\left( \mathbf{y }_h|\sigma ^2, \varvec{{w}}_h\right) \end{aligned}$$

For $k=2,\ldots ,H$ it follows:

$$\begin{aligned} \lambda _k^{-1}|\left( \mathbf{y} ,\varvec{{w}},\sigma ^2,\lambda _u, \{\lambda _h\}_{h\ne k}\right)\sim \;& {} GAM\left( \alpha _c + \frac{(k+1)}{2}, \beta _c + \frac{1}{2} \sigma ^{-2} D_k^2\right) \\ \text{ and }\;\;\;\lambda _u^{-1}|\left( \mathbf{y} ,\varvec{{w}},\sigma ^2,\{\lambda _h\}_{h\ge 2}\right)\sim \; & {} GAM\left( \alpha _u + \frac{(k+1)}{2}, \beta _u + \frac{1}{2} \sigma ^{-2} D_u^2 \right) \end{aligned}$$

where $$D_u^2 := \varvec{{w}}_{1}^{\mathsf{T}} \varvec{{w}}_{1}$$ and $$D_k^2 := (\varvec{{w}}_{k}- \tilde{\varvec{{w}}}_{k-1})^{\mathsf{T}} (\varvec{{w}}_{k}- \tilde{\varvec{{w}}}_{k-1})$$.

For each $\varvec{\theta }=\{\lambda _u,\{\lambda _h\}_{h\ge 2}\}$ the marginal likelihood, $p(\mathbf{y }|\{\lambda _u,\{\lambda _h\}_{h\ge 2}\})$, can be computed with Eq. (7); using the expressions $\mathbf{C} _h(\varvec{\theta })$ and $\Delta ^2(\varvec{\theta })$ defined above.

Unlike the proposed partially coupled M3 model, the generalized coupled M4 model does not feature any mechanism to uncouple neighbouring segments. Like the fully coupled M2 model, the M4 model has been designed such that it has to couple all neighbouring segments. The only advantage over the M2 model is that the the M4 model introduces segment-specific coupling parameters, so that the coupling strength(s) can vary over time.

### Reversible jump Markov chain Monte Carlo inference

We use Reversible Jump Markov Chain Monte Carlo simulations to generate posterior samples $\{\varvec{\pi }^{(w)},\varvec{\tau }^{(w)},\varvec{\theta }^{(w)}\}_{w=1,\ldots ,W}$. In each iteration we re-sample the parameters in $\varvec{\theta }$ from their full conditional distributions (Gibbs sampling), and we perform two Metropolis-Hastings moves; one on the covariate set $\varvec{\pi }$ and one on the changepoint set $\varvec{\tau }$. For the four models (M1–M4) Eq. (1) takes the form:

$$\begin{aligned} p\left( \varvec{\pi },\varvec{\tau },\varvec{\theta }|\mathbf{y }\right) \propto {\left\{ \begin{array}{ll} p(\varvec{\pi }) p(\varvec{\tau }) p\left( \lambda _u\right) \cdot p\left( \mathbf{y }|\varvec{\pi },\varvec{\tau },\lambda _u\right) &{}\quad \text{ M1 } \\ p(\varvec{\pi }) p(\varvec{\tau }) p\left( \lambda _u\right) \cdot p\left( \lambda _c\right) \cdot p\left( \mathbf{y }|\varvec{\pi },\varvec{\tau },\lambda _u,\lambda _c\right) &{}\quad \text{ M2 } \\ p(\varvec{\pi }) p(\varvec{\tau }) p\left( \lambda _u\right) \cdot p\left( \lambda _c\right) \cdot p\left( \{\delta _h\}_{h\ge 2}\right) \cdot p\left( \mathbf{y }|\varvec{\pi },\varvec{\tau },\lambda _u,\lambda _c,\{\delta _h\}_{h\ge 2} \right) &{}\quad \text{ M3 } \\ p(\varvec{\pi }) p(\varvec{\tau }) p\left( \lambda _u\right) \cdot \left( \prod _{h=2}^H p\left( \lambda _h\right) \right) \cdot p\left( \mathbf{y }|\varvec{\pi },\varvec{\tau },\lambda _u,\{\lambda _h\}_{h\ge 2} \right) &{}\quad \text{ M4 } \end{array}\right. } \end{aligned}$$

All likelihood terms, $p(\mathbf{y }|\ldots )$, are marginalized over $\sigma ^2$ and $\{\varvec{{w}}_h\}$ and for the new M3 model also the Bernoulli parameter $\text{ p }$ has been integrated out.

For the models M1–M2 the dimension of $\varvec{\theta }$ does not depend on $\varvec{\tau }$, while for the models M3–M4 the dimension of $\varvec{\theta }$ *does* depend on $\varvec{\tau }$. The M3 model has a discrete parameter $\delta _h\in \{0,1\}$ and the M4 model has a continuous parameter $\lambda _h\in \mathbb {R}^+$ for each $h>1$.

The model-specific full conditional distributions for the Gibbs sampling steps have been provided above. For sampling $\varvec{\pi }$ we implement 3 moves: covariate ‘removal (R)’, ‘addition (A)’, and ‘exchange (E)’. Each move proposes to replace $\varvec{\pi }$ by a new covariate set $\varvec{\pi }^{*}$ having one covariate more (A) or less (R) or exchanged (E). When randomly selecting the move type and the involved covariate(s), we get for all models the acceptance probability:

$$\begin{aligned}&A\left( \varvec{\pi }\rightarrow \varvec{\pi }^{*}\right) = \min \left\{ 1,\frac{p\left( \mathbf{y }|\varvec{\pi }^{*},\ldots \right) }{p\left( \mathbf{y }|\varvec{\pi },\ldots \right) } \cdot \frac{p\left( \varvec{\pi }^{*}\right) }{p(\varvec{\pi })} \cdot HR_{\varvec{\pi }}\right\} \\&\quad \text{ with } \text{ the } \text{ Hastings } \text{ Ratios: } \quad HR_{\varvec{\pi },R}=\frac{|\varvec{\pi }|}{n-|\varvec{\pi }^{*}|} , \quad HR_{\varvec{\pi },A}=\frac{n-|\varvec{\pi }|}{|\varvec{\pi }^{*}|}, \quad HR_{\varvec{\pi },E}=1 \end{aligned}$$

For sampling $\varvec{\tau }$ we also implement 3 move types: changepoint ‘birth (R)’, ‘death (D)’, and ‘re-allocation (R)’ moves. Each move proposes to replace $\varvec{\tau }$ by a new changepoint set $\varvec{\tau }^{*}$ having one changepoint added (B) or deleted (D) or re-allocated (R). When randomly selecting the move type, the involved changepoint and the new changepoint location, we get for M1 and M2:

$$\begin{aligned} A\left( \varvec{\tau }\rightarrow \varvec{\tau }^{*}\right)= & {} \min \left\{ 1,\frac{p\left( \mathbf{y }|\varvec{\tau }^{*},\ldots \right) }{p\left( \mathbf{y }|\varvec{\tau },\ldots \right) } \cdot \frac{p\left( \varvec{\tau }^{*}\right) }{p(\varvec{\tau })} \cdot HR_{\varvec{\tau }}\right\} \\&\text{ where } \quad HR_{\varvec{\tau },B}=\frac{T-1-|\varvec{\tau }|}{|\varvec{\tau }^*|}, \quad HR_{\varvec{\tau },D}=\frac{|\varvec{\tau }| }{T-1-|\varvec{\tau }^*|}, \quad HR_{\varvec{\tau },R}=1 \end{aligned}$$

For the models M3 (proposed here) and the model M4 from [6] the changepoint moves also affect the numbers of parameters in $\{\delta _h\}_{h\ge 2}$ and $\{\lambda _h\}_{h\ge 2}$, respectively. For all segments that stay identical we keep the parameters unchanged. For all new segments we re-sample the corresponding parameters. For the new model M3 we flip coins to get candidates for the involved $\delta _h$’s. This yields:

$$\begin{aligned} A\left( \left[ \varvec{\tau },\left\{ \delta _h\right\} \right] \rightarrow \left[ \varvec{\tau }^{*},\{\delta _h\}^{*}\right] \right) = \min \left\{ 1,\frac{p\left( \mathbf{y }|\varvec{\tau }^{*},\{\delta _h\}^{*},\ldots \right) }{p\left( \mathbf{y }|\varvec{\tau },\{\delta _h\},\ldots \right) } \frac{p\left( \varvec{\tau }^{*}\right) }{p(\varvec{\tau })} \frac{p\left( \{\delta _h\}^{*}\right) }{p\left( \left\{ \delta _h\right\} \right) } \cdot HR_{\varvec{\tau }} \cdot c_{\varvec{\tau }}\right\} \end{aligned}$$

where $c_{\varvec{\tau },B}=2$ for birth, $c_{\varvec{\tau },D}=1/2$ for death, and $c_{\varvec{\tau },R}=1$ for re-allocation moves. For the model M4 we follow [6] and re-sample the involved $\lambda _h$’s from their priors $p(\lambda _h)$. We obtain:

$$\begin{aligned} A\left( \left[ \varvec{\tau },\left\{ \lambda _h\right\} \right] \rightarrow \left[ \varvec{\tau }^{*},\{\lambda _h\}^{*}\right] \right) = \min \left\{ 1,\frac{p\left( \mathbf{y }|\varvec{\tau }^{*},\{\lambda _h\}^{*},\ldots \right) }{p\left( \mathbf{y }|\varvec{\tau },\{\lambda _h\},\ldots \right) } \cdot \frac{p\left( \varvec{\tau }^{*}\right) }{p(\varvec{\tau })} \cdot HR_{\varvec{\tau }} \right\} \end{aligned}$$

Note that the additional factor $c_{\varvec{\tau }}:=\frac{p(\{\lambda _h\})}{p(\{\lambda _h\}^{*})}$ of the Hastings ratio has been canceled with the prior ratio $\frac{p(\{\lambda _h\}^{*})}{p(\{\lambda _h\})}$.

### Edge scores and areas under precision recall curves (AUC)

For a network with *N* variables $Z_1,\ldots ,Z_N$ we infer *N* separate regression models. For each $Z_i$ we get a sample $\{\varvec{\pi }_i^{(w)},\varvec{\tau }_i^{(w)},\varvec{\theta }_i^{(w)}\}_{w=1,\ldots ,W}$ from the *i*th posterior. From the covariate sets we form a sample of graphs $G^{(w)}=\{\varvec{\pi }_{1}^{(w)},\ldots ,\varvec{\pi }_{N}^{(w)}\}_{w=1,\ldots ,W}$. For each edge $Z_i\rightarrow Z_j$ the edge posterior probability (edge score) is:

$$\begin{aligned} \hat{e}_{i,j}=\frac{1}{W} \sum _{w=1}^W I_{i\rightarrow j}\left( \mathcal {G}^{(w)}\right) \quad \text{ where } \quad I_{i\rightarrow j}\left( \mathcal {G}^{(w)}\right) = {\left\{ \begin{array}{ll} 1 &{}\quad \text{ if } X_i\in \varvec{\pi }_j^{(w)} \\ 0 &{}\quad \text{ if } X_i\notin \varvec{\pi }_j^{(w)} \end{array}\right. } \end{aligned}$$

If the true network is known and has *M* edges, we can quantify the network reconstruction accuracy. For each threshold $\xi \in [0,1]$ we extract the $n_{\xi }$ edges whose scores $\hat{e}_{i,j}$ exceed $\xi$, and we count the number of true positives $T_{\xi }$ among them. Plotting the precisions $P_{\xi }:=T_{\xi }/n_{\xi }$ against the recalls $R_{\xi }:=T_{\xi }/M$, gives the precision-recall curve. We refer to the area under the curve as AUC value.

### Hyperparameter settings and simulation details

The hyperparameters of the priors and hyperpriors of the four NH-DBN models (M1–M4) have to be specified in advance, and we note that the hyperparameter setting can have an effect on the resulting posterior distributions and so on the network reconstruction results. Selecting appropriate hyperparameters is therefore a crucial task. In the absence of genuine prior knowledge (e.g. from experts or from the literature), we re-use the rather uninformative (and thus generic) parameter settings from earlier publications. Re-using those hyperparameters also has the advantage that our empirical results can be compared with earlier reported results. More specifically, we proceed as follows:

For the models M1, M2 and M4 we re-use the hyperparameters from the earlier works by Lèbre et al. [1], Grzegorczyk and Husmeier [5], and Shafiee Kamalabad and Grzegorczyk [6]: $\sigma ^{-2}\sim GAM(\alpha _{\sigma }=\nu ,\beta _{\sigma }=\nu )$ with $\nu =0.005$, $\lambda _u^{-1}\sim GAM(\alpha _{u}=2,\beta _{u}=0.2)$, and $\lambda _c^{-1} \sim GAM(\alpha _{c}=3,\beta _{c}=3)$. For the new partially coupled model M3 we use the same setting with the extension: $\delta _h\sim BER(\text{ p})$ with $\text{ p }\sim BETA(a=1,b=1)$, which seems to be a very natural choice. For the M3 model we also tested several alternative hyperparameter settings, but we did not observe significantly deviating results, indicating that the M3 model is rather robust with respect to the hyperparameter settings. For more thorough studies on how the hyperparameter setting affects the network reconstruction results, we refer to the work by Grzegorczyk and Husmeier [5].

For all models M1–M4 we run each reversible jump Markov chain Monte Carlo simulation for $V=100{,}000$ iterations. Setting the burn-in phase to 0.5*V* (50%) and thinning out by the factor 10 during the sampling phase, yields $W=0.5V/10=5000$ samples from each posterior. To check for convergence, we compared the samples of independent simulations, using standard trace plot diagnostics as well as scatter plots of the estimated edge scores. For most of the data sets, analysed here, the diagnostics indicated almost perfect convergence already after $V=10{,}000$ iterations; see Fig. 7a for an example.

## Data

### Synthetic network data

For model comparisons we generated various synthetic network data sets. We report here on two studies with realistic network topologies, shown in Figs. 3 and 4. In both studies we assumed the data segmentation to be known. Hence, we kept the changepoints in $\varvec{\tau }$ fixed at their right locations and did not perform reversible jump Markov chain Monte Carlo moves on $\varvec{\tau }$.

*Study 1* For the RAF pathway with $N=11$ nodes and $M=20$ edges, shown in Fig. 4 and taken from Sachs et al. [16], we generated data with $H=4$ segments having $m=10$ data points each. For each node $Z_i$ and its parent nodes in $\varvec{\pi }_i$ we sampled the regression coefficients for $h=1$ from standard Gaussian distributions and collected them in a vector $\mathbf{w }^i_{1}$ which we normalised to Euclidean norm 1, $\mathbf{w }^i_{1}\leftarrow \mathbf{w }^i_{1}/|\mathbf{w }_{1}^i|$. For the segments $h=2,3,4$ we use: $\mathbf{w }^i_h = \mathbf{w }^i_{h-1}$ ($\delta _h=1$, coupled) or $\mathbf{w }^i_h = -\mathbf{w }^i_{h-1}$ ($\delta _h=0$, uncoupled). The design matrices $\mathbf{X }^i_h$ contain a first column of 1’s for the intercept and the segment-specific values of the parent nodes, shifted by one time point. To the segment-specific values of $Z_i$: $\mathbf{z }^i_h = \mathbf{X }^i_h \mathbf{w }^i_h$ we element-wise added Gaussian noise with standard deviation $\sigma = 0.05$. For all coupling scenarios $(\delta _2,\delta _3,\delta _4)\in \{0,1\}^3$, we generated 25 data sets having different regression coefficients.

*Study 2* This study is similar to the first one with three changes: (i) We used the yeast network with $N=5$ nodes and $M=8$ edges, shown in the left panel of Fig. 3 and taken from Cantone et al. [17]. (ii) Again we generated data with $H=4$ segments, but we varied the number of time points per segment $m\in \{2,3,\ldots ,12\}$. (iii) We focused on one scenario: For each node $Z_i$ and its parent nodes in $\varvec{\pi }_i$ we generated two vectors $\mathbf{w }^i_{\diamond }$ and $\mathbf{w }^i_{\star }$ with standard Gaussian distributed entries. We re-normalised the first vector to Euclidean norm 1, $\mathbf{w }^i_{\diamond }\leftarrow \mathbf{w }^i_{\diamond }/|\mathbf{w }^i_{\diamond }|$, and the 2nd vector to norm 0.5, $\mathbf{w }^i_{\star }\leftarrow 0.5\cdot \mathbf{w }^i_{\star }/|\mathbf{w }^i_{\star }|$. We set $\mathbf{w }^i_1= \mathbf{w }^i_2 = \mathbf{w }^i_{\diamond }$ so that the segments $h=2$ and $h=3$ are coupled, and $\mathbf{w }^i_3= \mathbf{w }^i_4 = (\mathbf{w }^i_{\diamond }+\mathbf{w }^i_{\star })/(|\mathbf{w }^i_{\diamond }+\mathbf{w }^i_{\star }|)$, so that the segments $h=3$ and $h=4$ are coupled, while the coupling between $h=3$ and $h=2$ is ‘moderate’. For each *m* we generated 25 data matrices with different regression coefficients.

### Yeast gene expression data

Cantone at al. [17] synthetically designed a network in *S. cerevisiae* (yeast) with $N=5$ genes, and measured gene expression data under galactose- and glucose-metabolism: 16 measurements were taken in galactose and 21 measurements were taken in glucose, with 20 minutes intervals in between measurements. Although the network is small, it is an ideal benchmark data set: The network structure is known, so that network reconstruction methods can be cross-compared on real wet-lab data. We follow Grzegorczyk and Husmeier and pre-process the data as described in [5]. The true network structure is shown in the left panel of Fig. 3. As an example, a network prediction obtained with the partially coupled model (M3) is shown in the right panel. For the prediction we extracted the 8 edges with the highest scores.

### Arabidopsis gene expression data

The circadian clock in *Arabidopsis thaliana* optimizes the gene regulatory processes with respect to the daily dark:light cycles (photo periods). In four experiments Arabidopsis plants were entrained in different dark:light cycles, before gene expression data were measured under constant light condition over 24- and 48-h time intervals. We follow Grzegorczyk and Husmeier [5] and merge the four time series to one single data set with $T=47$ data points and focus our attention on the $N=9$ core genes: LHY, TOC1, CCA1, ELF4, ELF3, GI, PRR9, PRR5, and PRR3.

## Results

In this section we present the results of a comparative evaluation study, in which we compare the performance of the new partially coupled model (M3) with the competing models M1, M2 and M4. Throughout this section we use the new M3 model as reference model.

### Results for synthetic network data

We start with the RAF-pathway for which we generated network data for 8 different coupling scenarios. Figure 5a compares the network reconstruction accuracies in terms of average AUC value differences. For 6 out of 8 scenarios the three AUC differences are clearly and significantly in favour of M3. Not surprisingly, for the two extreme scenarios, where all segments $h\ge 2$ are either coupled (‘0111’) or uncoupled (‘0000’), M3 performs slightly worse than the fully coupled models (M2 and M4) or the uncoupled model (M1), respectively. But unlike the uncoupled model (M1) for coupled data (‘0111’), and unlike the coupled models (M2 and M4) for uncoupled data (‘0000’), the partially coupled model (M3) never performs significantly worse than the respective ‘gold-standard’ model. For the partially coupled model, Fig. 5b shows the posterior probabilities that the segments $h=2,3,4$ are coupled. The trends are in good agreement with the true coupling mechanism. Model M3 correctly infers whether the regression coefficients stay similar (identical) or change (substantially). The generalised coupled model (M4) can only adjust the segment-specific coupling strengths, but has no option to uncouple. Like the coupled model (M2), it fails when the parameters are subject to drastic changes. When comparing the coupled model (M2) with the generalised coupled model (M4), we see that M2 performs better when only one segment is coupled, while the new M4 model is superior to M2 if two segments are coupled, see the scenarios ‘0011’, ‘0110’, and ‘0101’.

**Fig. 6:**
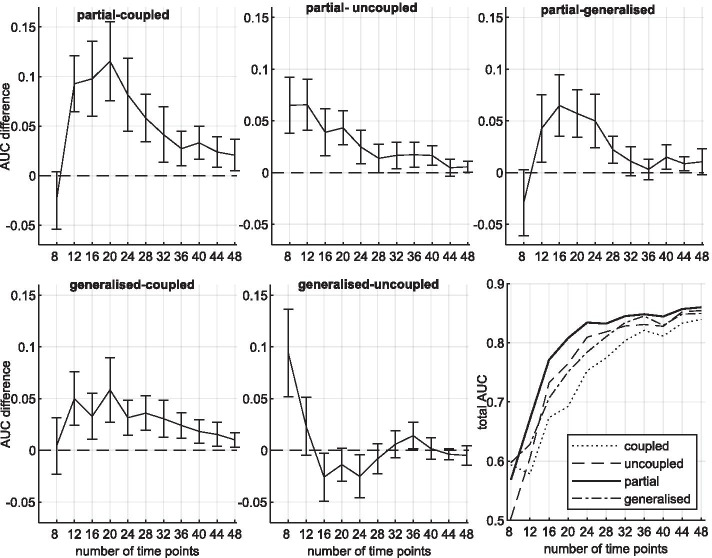
Results for synthetic yeast data, generated under coupling scenario (0, 1, 1, 1). Five panels show the average AUC differences plotted against the numbers of data points *T*. The error bars indicate *t* test confidence intervals. The bottom right panel shows the model-specific average AUC values

**Fig. 7:**
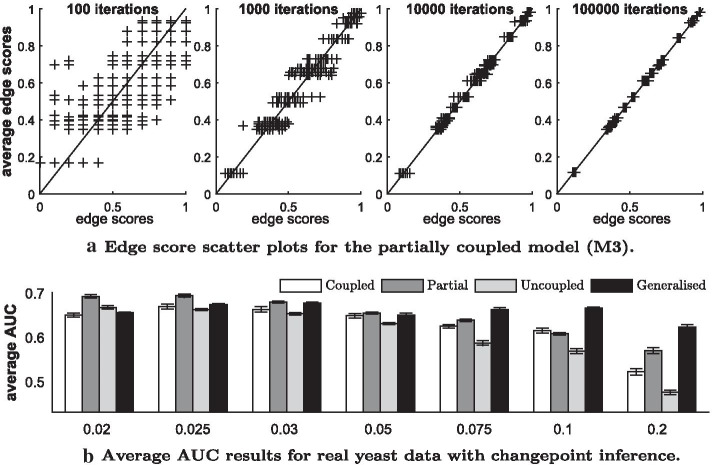
Analysis of the real yeast data. (**a**) For each run length, $V\in \{100,1000,10{,}000,100{,}000\}$ we performed 15 RJMCMC simulations with the partially coupled model (M3). We used the hyperparameter $p=0.05$ for the changepoint prior. For each *V* there is a scatter plot where the simulation-specific edge scores (vertical axis) are plotted against the average scores for that *V* (horizontal axis). (**b**) We implemented the models M1–M4 with different hyperparameters *p* of the geometric distribution for the distance between changepoints. For each *p* the bars show the model-specific average AUC scores. The error bars indicate standard deviations

**Fig. 8:**
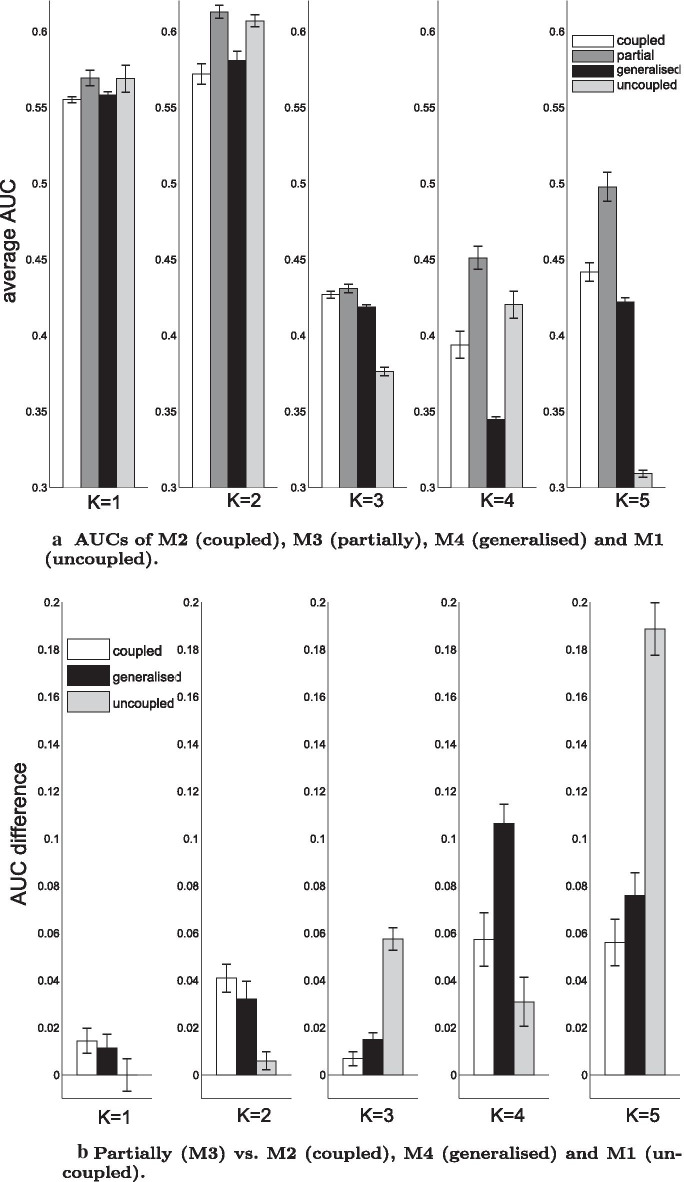
Results for real yeast data with fixed changepoints. We imposed $K\in \{1,\ldots ,5\}$ changepoints and kept them fixed. *K* changepoints yield $H=K+1$ segments. For each *K* we used the first changepoint to separate the two parts of the time series (galactose vs. glucose metabolism). Successively we located the next changepoint in the middle of the longest segment to divide it into 2 segments, until *K* changepoints were set. **a** show the model-specific average total AUC scores with error bars indicating standard deviations. **b** shows the AUC score differences in favour of the partially coupled model (M3). Here the error bars indicate t-test confidence intervals

**Fig. 9:**
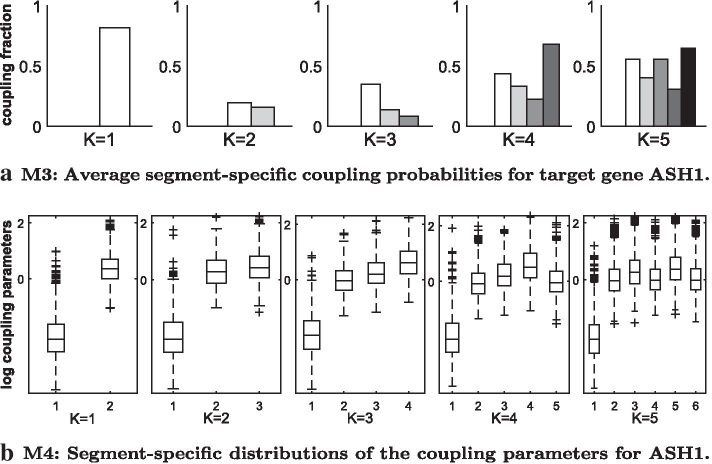
Results for real yeast data with fixed changepoints. We imposed $K\in \{1,\ldots ,5\}$ changepoints and kept them fixed. *K* changepoints yield $H=K+1$ segments. For each *K* we used the first changepoint to separate the two parts of the time series (galactose vs. glucose metabolism). Successively we located the next changepoint in the middle of the longest segment to divide it into 2 segments, until *K* changepoints were set. **a** Diagnostic for the partially coupled model (M3): The bars give the posterior probabilities $p(\delta _h=1|\mathcal {D})$ that segment *h* is coupled to $h-1$ ($h=2,\ldots ,K+1$) for target gene ASH1. **b** Diagnostic for the generalised coupled model (M4): In each panel there is a boxplot for each segment $h=2,\ldots ,K+1$ showing the distributions of the logarithmic coupling parameters $\lambda _h$ for target gene ASH1

For the yeast network we generated data corresponding to a ‘0101’ coupling scheme and the change of the parameters (from the 2nd to the 3rd segment) is less drastic than for the RAF pathway data. Figure 6 shows how the AUC differences vary with the number of time points *T*, where $T=4m$ and *m* is the number of data points per segment. For sufficiently many data points the effect of the prior diminishes and all models yield high AUC values (see bottom right panel). There are then no significant differences between the AUC values anymore. However, for the lower sample sizes again the new partially coupled model (M3) performs clearly best. For $12 \le m \le 28$ model M3 is significantly superior to all other models and for $30 \le T \le 40$ it still significantly outperforms the uncoupled (M1) and the coupled (M2) model. The performance of the generalised model (M4) is comparable to the performance of the uncoupled model. For moderate sample sizes ($12 \le T \le 44$) model M4 is significantly better than the fully coupled model (M2).

### Results for yeast gene expression data

For the yeast gene expression data we assume the changepoint(s) to be unknown and we infer the segmentation from the data. Figure 7a shows convergence diagnostics for the partially coupled model (M3). It can be seen from the scatter plots that $V=10{,}000$ RJMCMC iterations yield already almost perfect convergence. The edge scores of 15 independent MCMC runs are almost identical to each other.

The average AUC scores of the models M1–M4 are shown in Fig. 7b. Since the number of inferred changepoints grows with the hyperparameter *p* of the geometric distribution on the distance between changepoints, we implemented the models with different *p*’s. The uncoupled model is superior to the coupled model for the lowest *p* ($p=0.02$) only, but becomes more and more inferior to the coupled model, as *p* increases. This result is consistent with the finding in Grzegorczyk and Husmeier [5] and can be explained as follows: As the hyperparameter of the changepoint prior $p\in (0,1)$ increases, the number of inferred data segments *H* grows so that the individual data segments $h=1,\ldots,H$ get shorter. The individual segments *h* then cover less data points and are thus less informative. The coupling scheme allows for information-sharing among segments. The information content of large segments is sufficient for inference, so that coupling does not provide any noteworthy advantage. But for short (uninformative) segments information coupling improves the inference certainty, as coupling allows for the incorporation of information from the preceding segment(s). Therefore the potential improvement that can be gained by coupling grows with the hyperparameter *p*.

The new partially coupled model (M3) performs consistently better than the uncoupled and the coupled model (M1–M2). The only exemption occurs for $p=0.1$ where the coupled model (M2) appears to perform slightly (but not significantly) better than M3. For *p*’s up to $p=0.05$ the fully coupled (M2) and the generalised fully coupled model (M4) perform approximately equally well. However, for the three highest *p*’s the M4 model performs better than the coupled model (M2) and even outperforms the new partially coupled model (M3). While the performances of the models M1–M3 decrease with the number of changepoints, the performance of the model M4 stays rather robust.

Subsequently, we re-analysed the yeast data with $K=1,\ldots ,5$ fixed changepoints. Figure 8a, b shows the average AUC scores and the AUC score differences in favour of the partially coupled model (M3). Panel (a) reveals that the new partially coupled model (M3) reaches again the highest network reconstruction accuracy. Panel (b) shows that the superiority of M3 is significant, with only one exemption: For $K=1$ the uncoupled model M1 does not perform worse than the partially coupled model (M3).

Subsequently, we also investigated the segment-specific coupling posterior probabilities $p(\delta _h=1|\mathcal {D})$ ($h=2,\ldots ,H=K+1$) for the new partially coupled model (M3) and the posterior distributions of the coupling parameters $\lambda _u,\lambda _2,\ldots ,\lambda _{K+1}$ for the generalised model (M4), but we could not find clear trends for any gene. As an example, we provide the results for gene ASH1 in Fig. 9a, b. Panel (a) shows that the coupling posterior probabilities of model M3 do not have a clear pattern. However, it becomes obvious that the partially coupled model makes use of segment-wise switches between the uncoupled and the coupled approach. Panel (b) shows that the distributions of the segment-specific coupling parameters, $\lambda _2,\ldots ,\lambda _{K+1}$, of model M4 stay rather similar among segments. This explains why the generalised coupled model (M4) is not superior to the fully coupled model (M2).

### Application to Arabidopsis gene expression data

**Fig. 10:**
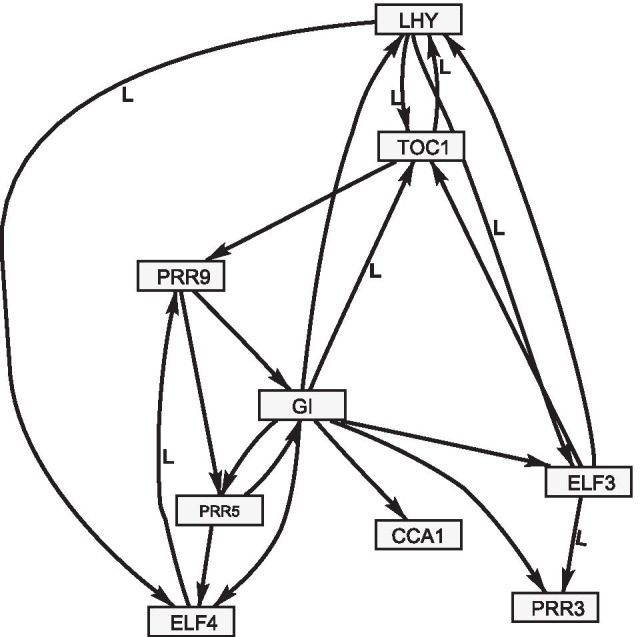
Prediction of the circadian clock network in *Arabidopsis thaliana*. The prediction was obtained with the proposed partially coupled model (M3), using the hyperparameter $p=0.1$ for the geometric distribution on the distance between changepoints. The network shows the 20 edges with the highest edge scores. We have added the label ‘L’ to those edges that have already been reported in the biology literature. Fore more details see the main text

For the Arabidopsis gene expression data we cannot objectively compare the network reconstruction accuracies of the four models, since the true circadian clock network is not known. We therefore only applied the new partially coupled model (M3), which we had found to be the best model in our earlier studies. Figure 10 shows the Arabidopsis network, which was reconstructed using the hyperparameter $p=0.1$ for the geometric distribution on the distance between changepoints. To obtain a network prediction, we extracted the 20 edges with the highest edge scores. Although a proper evaluation of the network prediction is beyond the scope of this paper, we note that several features of the network are consistent with the plant biology literature. E.g. the feedback loop between *LHY* and *TOC*1 is the most important key feature of the circadian clock network (see, e.g., the work by Locke et al. [18]). Many of the other predicted edges have been reported in more recent works. E.g. the edges $LHY\rightarrow ELF3$, $LHY\rightarrow ELF4$, $GI\rightarrow TOC1$, $ELF3\rightarrow PRR3$ and $ELF4\rightarrow PRR9$ can all be found in the circadian clock network (hypothesis) of Herrero et al. [19].

## Discussion and conclusions

We have proposed a new Bayesian piece-wise linear regression model for reconstructing regulatory networks from gene expression time series. The new partially coupled model (M3), whose graphical model representation is given in Fig. 2, is a consensus model between the uncoupled model (M1) and the fully coupled model (M2). In the uncoupled model (M1) the segment-specific regression coefficients have to be learned for each segment separately. In the fully coupled model (M2) each segment is compelled to be coupled to the previous one. The new partially coupled model (M3) combines features of the uncoupled and the fully coupled model, and it can infer for each individual time segment whether it is coupled to (or uncoupled from) the preceding segment.

We have cross-compared the new model (M3) with the two established models (M1–M2) as well as with the generalised coupled model (M4) that makes use of segment-specific coupling parameters [6]. In our data applications, the new partially coupled model (M3) reached significantly better network reconstruction accuracies than its competitors (M1, M2, and M4).

In an earlier work [6], we found that the performances of the fully coupled model (M1) and of the generalised fully coupled model (M4) can be improved by imposing additional hyperpriors on the hyperparameters of the coupling strength parameter. In our future work we will therefore investigate whether either the use of hyperpriors or the use of segment specific continuous (coupling/SNR) parameters along the lines of the M4 model can improve the new partially coupled model (M3). Moreover, in our future work we will also try to combine the concept of partially coupled time segments of the proposed model (M3) with the recently proposed concept of partially coupled edges [8]. The combination of both concepts will yield a highly flexible novel NH-DBN model, in which each individual network edge is partially segment-wise coupled. We will empirically test whether this new hybrid model leads to improved network reconstruction results or whether it suffers from model over-flexibility.

## Supplementary information

**Additional file 1**. Graphical model representations of the three competing models are provided as additional files. Figure 11 shows a graphical model representation of the M1 model. Figure 12 shows a graphical model representation of the M2 model. Figure 13 shows a graphical model representation of the M4 model.

## Data Availability

The datasets analysed during the current study are available in the figshare repository, https://figshare.com/s/96f578777aa6b43f3638 We note that the data stem from earlier publications [5, 17].

## Abbreviations

DBN: Dynamic Bayesian network
NH-DBN: Non-homogeneous dynamic Bayesian network
MCMC: Markov chain Monte Carlo
RJMCMC: Reversible jump Markov chain Monte Carlo
SNR: Signal-to-noise ratio
AUC: Areas under precision recall curve
