# Supplementary figures and images for: A new Bayesian piecewise linear regression model for dynamic network reconstruction
Source: BMC Bioinformatics. 2021 Apr 26;22(Suppl 2):196. doi: 10.1186/s12859-021-03998-9 (PMC8074473; doi:10.1186/s12859-021-03998-9)

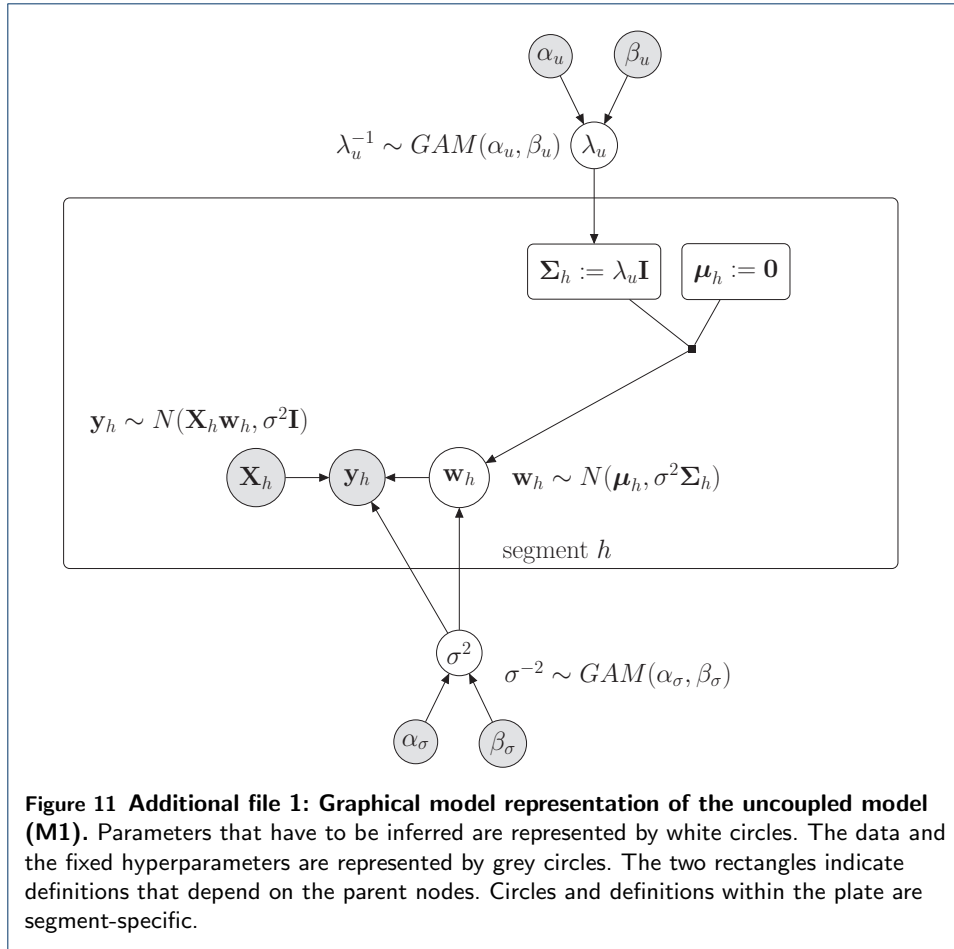

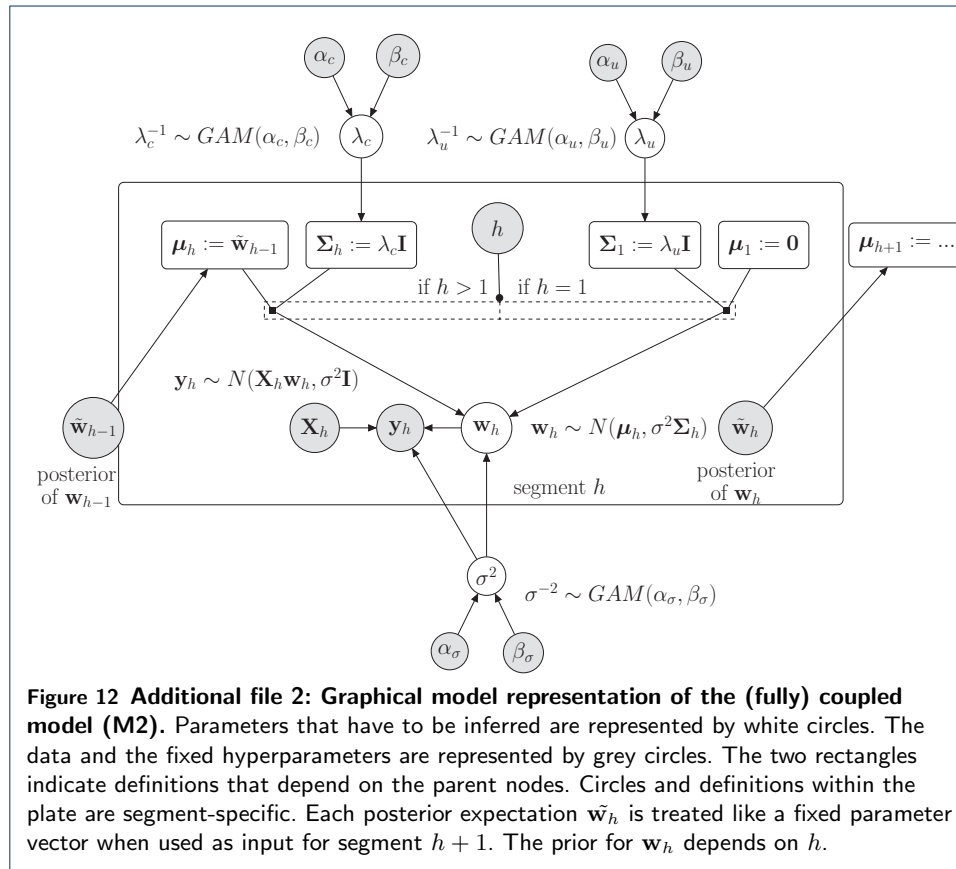

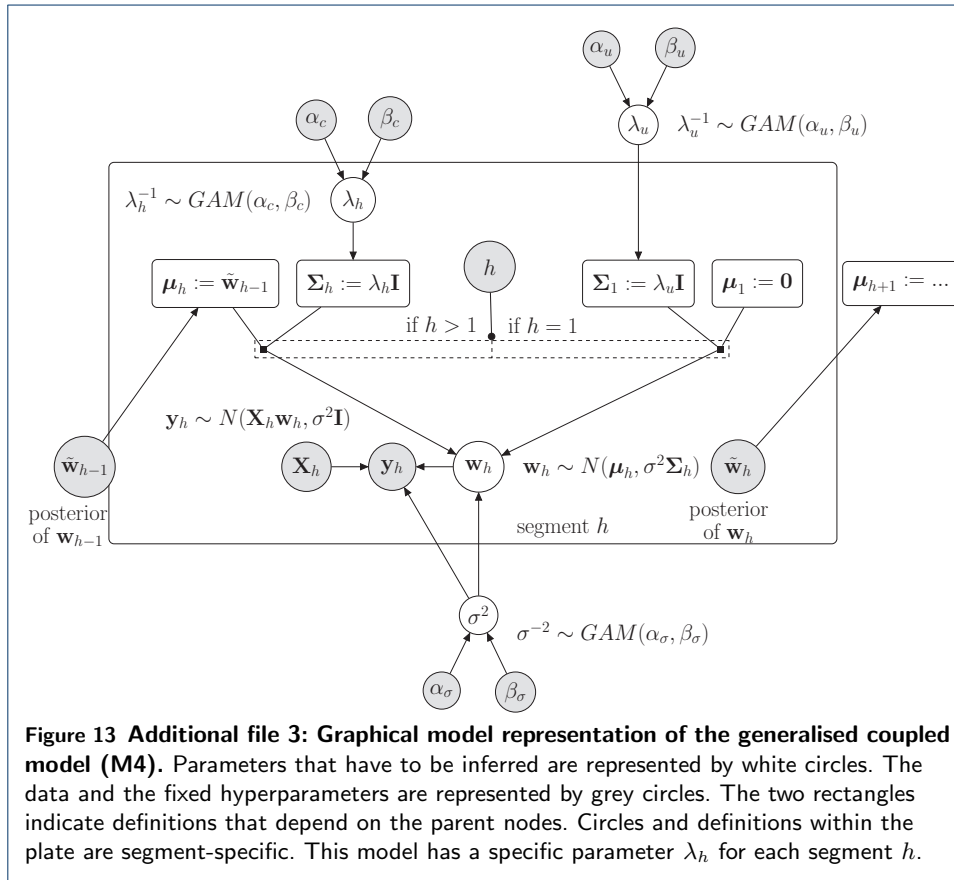

Supplement: Supplementary file 1 — Additional file 1. Graphical model representations of the three competing models are provided as additional files. Figure 11 shows a graphical model representation of the M1 model. Figure 12 shows a graphical model representation of the M2 model. Figure 13 shows a graphical model representation of the M4 model. [file 12859_2021_3998_MOESM1_ESM.pdf]
